# Clinical Experience for Modified Thoracoabdominal Nerve Block Through Perichondrial Approach (M-TAPA) in Five Patients. Dermatomal Evaluation and Application of Different Volumes: A Case Series and Review of Literature

**DOI:** 10.4274/TJAR.2022.221042

**Published:** 2023-08-18

**Authors:** Bahadır Çiftçi, Hande Güngör, Selçuk Alver, Ayşe Nurmen Akın, Yaşar Özdenkaya, Serkan Tulgar

**Affiliations:** 1 Department of Anaesthesiology and Reanimation, İstanbul Medipol University, Mega Medipol University Hospital, İstanbul, Turkey; 2 Department of General Surgery, İstanbul Medipol University, Mega Medipol University Hospital, İstanbul, Turkey; 3 Department of Anaesthesiology and Reanimation, Samsun University Faculty of Medicine, Samsun Training and Research Hospital, Samsun, Turkey

**Keywords:** Abdominal surgery, dermatomal coverage, modified thoracoabdominal nerves block, postoperative analgesia, regional anaesthesia

## Abstract

Thoracoabdominal nerves block through perichondrial approach (TAPA) is a novel block and provides abdominal analgesia. TAPA block targets the both anterior and the lateral branches of the thoracoabdominal nerves. Modified-TAPA (M-TAPA) was defined due to the need for blocking certain dermatomes depending on the surgical incision sites. In the literature, the knowledge about the efficiency and dermatomal coverage of M-TAPA is limited. In this case series, we want to report our experiences with this issue.

**Main Points**

• Modified thoracoabdominal nerves block through perichondrial approach (M-TAPA) is a novel abdominal wall block and it targets anterior and the lateral branches of the thoracoabdominal nerves.

• In the literature, there are several case reports and studies about the analgesic efficacy of M-TAPA.

• However, there is still a need for the knowledge about the dermatomal coverage of M-TAPA.

## Introduction

Thanks to the use of ultrasound, novel plane blocks have been defined in recent years. Recently Tulgar et al.^1^ defined the thoracoabdominal nerves block through perichondrial approach (TAPA) for abdominal analgesia. TAPA block targets the both anterior and the lateral branches of the thoracoabdominal nerves. It provides effective analgesia in a large dermatomal area due to this mechanism of action. Following the description of TAPA, again Tulgar et al.^2^ redefined the TAPA block and named this novel technique as modified-TAPA (M-TAPA). They defined the M-TAPA block due to the need for blocking certain dermatomes depending on the surgical incision sites. They applied 50 mL of local anaesthetic (LA) (bupivacaine 0.25%) only to the lower surface of the perichondrium. After the first description of M-TAPA, it has been successfully used for several abdominal procedures such as ventral hernia repair, and laparoscopic sleeve gastrectomy.^3,4^ We have read with great interest the M-TAPA articles.^1,2,3,4^ However, the volumes of LA used are different in each case report. Fascial plane blocks are volume-related blocks, the efficacy may depend on the LA volume.^5^ Therefore, we decided to perform lower volume M-TAPA than in the literature for different laparoscopic abdominal surgeries.

## Case Presentation

**Figure 1:**
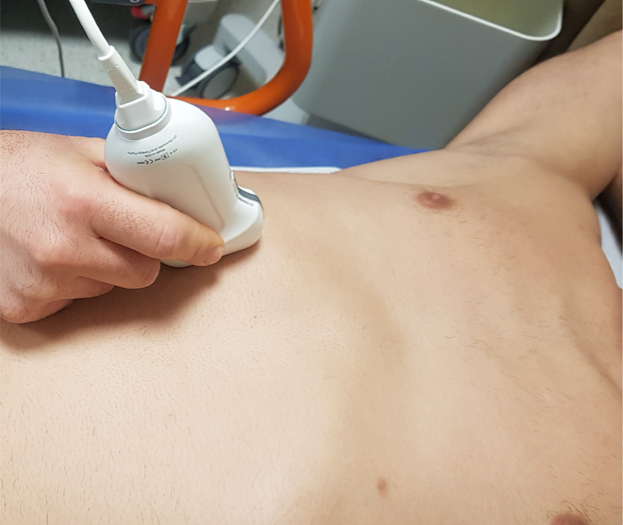
Patient position and probe placement during M-TAPA. Minimal compression from caudal to cranial over the probe may improve visualization. M-TAPA, modified-through perichondrial approach.

**Figure 2:**
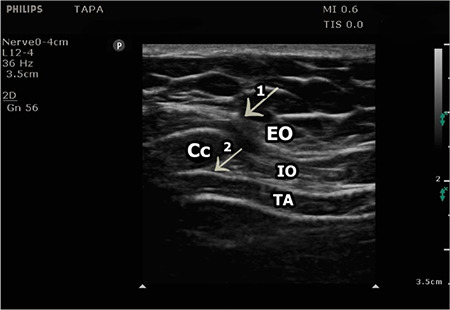
Sonographic anatomy of TAPA and M-TAPA. Arrows indicate the needle directions. 1 + 2 = TAPA, only 2 = M-TAPA. Cc, costal cartilage; EO, external oblique muscle; IO, internal oblique muscle; TA, transversus abdominis muscle, M-TAPA, modified-through perichondrial approach.

**Figure 3:**
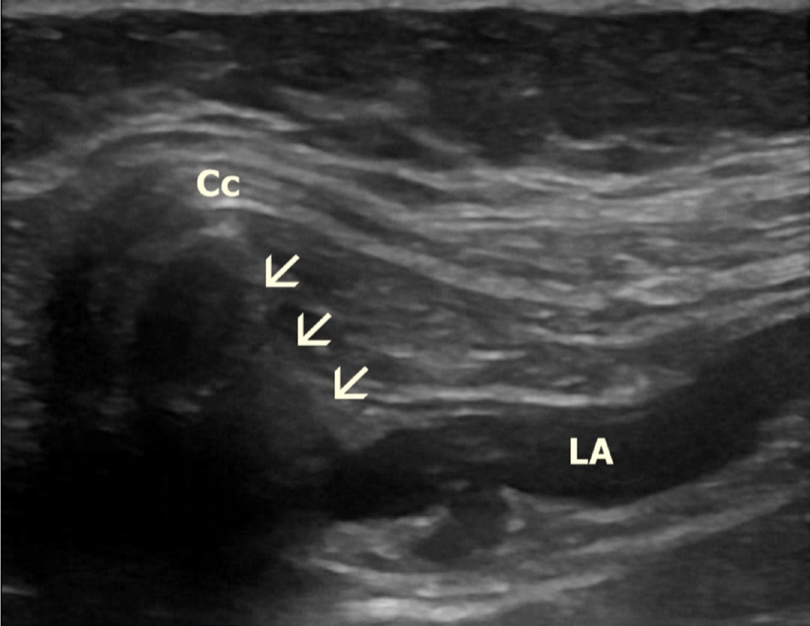
Sonographic visualization of LA. Arrow indicates the needle. Cc, costal cartilage; LA, local anesthetic.

We performed bilateral M-TAPA in our patients after the end of the surgery before extubation (Figure 1). A high-frequency linear transducer (11-12 MHz) was placed deep into the costochondrium at the level of the 9^th^-10^th^ ribs (Figures 2, 3). We used 0.25% bupivacaine for the block. We performed 400 mg ibuprofen and 100 mg tramadol on our patients 20 min before the end of the surgery. We evaluate the dermatomal area with a pin-prick test in our patients during the postoperative 1^st^ hour. We ordered a dose of 400 mg ibuprofen IV every 8 hours for the routine postoperative analgesia protocol. We evaluated pain scores with the numeric rating scale (NRS).

Case 1 was a 25-year-old female patient (165 cm, 61 kg) with no co-morbidity, who underwent laparoscopic cholecystectomy surgery. The operation was uneventful (surgery lasted 60 min), and her hemodynamic parameters were stable during the surgery. We performed bilateral M-TAPA with 15 + 15 mL for each side (30 mL total). NRS was <2 at the postoperative 24-hour period. No additional analgesia was needed. The dermatomal area was between T6-T12 dermatomes (Table 1).

Case 2 was a 53-year-old male patient (156 cm, 68 kg) with regulated hypertension, who underwent laparoscopic cholecystectomy surgery. The operation was uneventful (surgery lasted 65 min), and his hemodynamic parameters were stable during the surgery. We performed bilateral M-TAPA with 15 + 15 mL for each side (30 mL total). NRS was <1 at the postoperative 24-hour period. No additional analgesia was needed. The dermatomal area was between T6-T11 dermatomes (Table 1).

Case 3 was a 47-year-old male patient (170 cm, 82 kg) with no co-morbidity, who underwent laparoscopic cholecystectomy surgery. The operation was uneventful (surgery lasted 50 min), and his hemodynamic parameters were stable during the surgery. We performed bilateral M-TAPA with 15 + 15 mL for each side (30 mL total). NRS was <3 at the postoperative 24-hour period. No additional analgesia was needed. The dermatomal area was between T8-T12 dermatomes (Table 1).

Case 4 was a 46-year-old female patient (170 cm, 82 kg) with regulated hypothyroidism, who underwent laparoscopic incisional hernia repair surgery. The operation was uneventful (surgery lasted 180 min), and her hemodynamic parameters were stable during the surgery. We performed bilateral M-TAPA with 20 + 20 mL for each side (40 mL total). NRS was 0 during the postoperative 24-hour period. No additional analgesia was needed. The dermatomal area was between T6-T12 dermatomes (Table 1).

Case 5 was a 65-year-old male patient (185 cm, 79 kg) with regulated hypertension, who underwent laparoscopic inguinal hernia repair surgery. The operation was uneventful (surgery lasted 80 min), and his hemodynamic parameters were stable during the surgery. We performed bilateral M-TAPA with 20 + 20 mL for each side (40 mL total). NRS was <4 at the postoperative 24-hour period. No additional analgesia was needed. The dermatomal area was between T7-T11 dermatomes (Table 1).

The demographic data, pain scores, and dermatomal evaluation of the patients are shown in Table 1.

## Discussion

M-TAPA is a novel plane block and has been used successfully for several abdominal surgeries with its opioid-sparing effect. According to our case series presentation, there are nearly similar results with different volumes of LA. The NRS of our patients was low, however, the dermatomes were different.

In the first description of M-TAPA, Tulgar et al.^2^ performed 50 mL volume of LA bilaterally (25 mL for each side) for a patient who underwent laparotomy due to metastatic ovarian cancer. They performed M-TAPA just after the anesthesia induction. They reported that there was no need for extra analgesia during surgery, even though they stopped the infusion of remifentanil. They reported a dermatomal area that included T7-T11 dermatomes from the anterior axillary line to the midline bilaterally, and NRS scores <3/10. Altıparmak et al.^3^ performed M- TAPA with 40 mL of LA bilaterally for a patient who underwent laparoscopic ventral hernia repair. They preferred M-TAPA instead of TAPA due to technical difficulty performing TAPA. They reported that the NRS score of the patient was 2-3/10 at the postoperative 15^th^ and 30^th^ minutes. The dermatomal coverage was between T5-T10 levels at the postoperative 60^th^ min. de Oliveira et al.^6^ performed M-TAPA in 12 patients who underwent laparoscopic sleeve gastroplasty surgery. They used a total of 40 to 60 mL volume of LA (4 patients to 6, respectively). They evaluated pain scores and quality of recovery scores (QoR-15) after surgery. They reported that higher pain levels were observed in patients performed with 40 mL of LA. According to their case series, the QoR-15 scores were between moderate and excellent. The authors concluded that M-TAPA may be an alternative analgesia technique for the upper abdomen levels and the lateral wall of the abdomen. Aikawa et al.^4^ performed M-TAPA on a patient with co-morbidities who underwent laparoscopic sleeve gastrectomy surgery. The authors did not prefer epidural anesthesia due to previous thoracic spine surgery. They performed M-TAPA bilaterally with a 60 mL volume of LA (30 mL for each side). They reported that the patient had no pain and needed no extra analgesia during the postoperative period. They evaluated the dermatomal area between T3-T12 from the posterior axillary line to the midline. They reported that the effect of the sensorial block disappeared at 56 h after the block. Additionally, we can see different usage areas for M-TAPA in the literature. Balaban et al.^7^ performed M-TAPA for surgical anesthesia after failed erector spinae plane block (ESPB) in a patient who underwent pericholecystic drainage catheter placement. They reported that the patient had a chronic obstructive pulmonary disease and had cyanosis with 86% peripheral saturation. They performed M-TAPA with a 30 mL volume of LA (bupivacaine + lidocaine) for surgical anesthesia after the failure of ESPB. After 30 min from the M-TAPA procedure, they determined sensorial coverage between T6-T11. On the other hand, a continuous catheter may be used in M-TAPA. Ohgoshi et al.^8^ performed continuous M-TAPA in 2 patients who underwent adhesion surgery. They reported that patients had no pain during the postoperative period, and needed no extra analgesic.

Clinical research articles are limited to M-TAPA, yet. Bilge et al.^9^ compared M-TAPA vs no block control group in their prospective and randomized study. They concluded that M-TAPA reduced pain scores and opioid consumption during the postoperative period. They reported that the QoR-40 scores were higher in the M-TAPA group. Aikawa et al.^10^ performed a dermatomal analyses study on patients who underwent gynecological laparoscopic surgery. In their prospective observational surgery, they performed M-TAPA with a 25 mL volume of LA. They reported that the highest sensory level was T7 (T5-T8) in the anterior and T9 (T7-T10) in the lateral area. They did not observe a sensory loss in the lateral area in 5 patients. A recent dermatomal analyses study was performed by Ohgoshi et al.^11^ They compared external oblique muscle plane block (EXOP) vs M-TAPA in ten volunteers. They used a 20 mL volume of LA for each block. They concluded that M-TAPA anaesthetized only the anterior branches from T6/7 to T11/12, whereas EXOP anaesthetized the lateral cutaneous branches of T7-10 and T11-12. They concluded that the combined use of M-TAPA and EXOP may anaesthetize the entire abdominal wall. However, in our case series, we observed that there was a sensorial block in both the anterior and lateral abdominal walls.

Ciftci et al.^12^ performed a cadaveric investigation and they compared the spread of dye between TAPA and M-TAPA. They reported that there was dye on the thoracoabdominal nerves (T4-T12), and over external and internal oblique muscles. Additionally, they reported that the spread of M-TAPA over the transversus abdominis muscle [transversus abdominis plane (TAP)] was in a wider area than TAPA. Since the thoracoabdominal nerves run through the TAP, here is important for the mechanism of action. In our patients, M-TAPA provided adequate pain control and dermatomal coverage. In a recent prospective observational pilot study, Tanaka et al.^13^ evaluated the efficacy of M-TAPA in open gynecological surgery, and they performed a cadaveric evaluation. They reported that M-TAPA had dermatomal coverage in the areas supplied by the anterior branches of T8-T11. They observed dye spread between T8-T11 in both 2 cadavers.

## Conclusion

In our patients, M-TAPA provided adequate pain control and dermatomal coverage. Further studies and cadaveric examinations are needed to evaluate the exact mechanism and efficiency of M-TAPA. In summary, M-TAPA provides effective pain control after laparoscopic abdominal surgeries.

## Figures and Tables

**Table 1:**
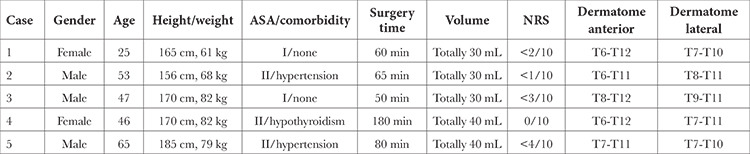
The Demographic Data, Pain Scores, and Dermatomal Evaluation of the Patients
